# The Safety of OnabotulinumtoxinA (OnabotA) During Pregnancy and Breastfeeding: Implications for Chronic Migraines

**DOI:** 10.3390/life16081228

**Published:** 2026-07-24

**Authors:** Sondos Eladawi, Prut Koonalintip, Amelia Ngomuo, Emily Leech, Mark Thaller, Benjamin R. Wakerley

**Affiliations:** 1 Department of Neurology, Queen Elizabeth Hospital Birmingham, University Hospitals Birmingham NHS Foundation Trust, Mindelsohn Way, Edgbaston, Birmingham B15 2GW, UK; sondos.eladawi@nhs.net (S.E.);; 2 Division of Neurology, Department of Internal Medicine, Prince of Songkla University, Hatyai 90110, Songkhla, Thailand; 3 Birmingham Medical School, College of Medicine and Health, University of Birmingham, Edgbaston, Birmingham B15 2TT, UK; 4 Institute of Metabolism and Systems Research, College of Medicine and Health, University of Birmingham, Edgbaston, Birmingham B15 2TT, UK; 5 Department of Neurology, Sandwell and West Birmingham NHS Trust, West Bromwich B71 4HJ, UK

**Keywords:** chronic migraines, dystonia, pregnancy, OnabotulinumtoxinA

## Abstract

Background and objectives: A migraine is a highly prevalent and disabling neurological disorder that mainly affects women of childbearing age. Treatment options for migraine prevention are limited during pregnancy and breastfeeding. Although OnabotulinumtoxinA (OnabotA) has proven efficacy and safety in chronic migraine prophylaxis in adults, its safety in pregnancy and breastfeeding remains understudied. Methods: Using the Preferred Reporting Items for Systematic Reviews and Meta-Analyses methodology, this review systematically appraised the evidence on the use of OnabotA for chronic migraines and other neurological conditions during pregnancy and breastfeeding across four electronic databases between January 2000 and December 2025. Results: In summary, of the 555 OnabotA-exposed pregnancies, 80.2% resulted in full-term births, with a 17.5% loss rate (73 spontaneous, 21 elective, three unspecified) and 1.6% reporting foetal anomalies. These findings align with general population baselines for miscarriages (12.5–18.7%) and foetal anomalies (3–5%), respectively. Among 94 cases of reported breastfeeding, no adverse infant events or developmental delays were observed at 1 year. Maternal outcomes showed favourable symptom control without adverse effects. Conclusions: Although there is limited data on the use of OnabotA in pregnancy and breastfeeding, no significant adverse effects have been identified in pregnant mothers or neonates. OnabotA should therefore be considered on a case-by-case basis for treatment of various neurological conditions, including migraines, when there are no better safe alternatives.

## 1. Introduction

Migraines are among the most prevalent neurological disorders worldwide and a leading cause of disability [1]. Migraines are especially common in women of childbearing age, and almost a third will experience an attack before the age of 45 years old [1]. Migraine attacks are characterised by a moderate-to-severe headache, which is often associated with nausea, sensitivity to light and noise, and frequently reduced ability to function. Approximately a third of patients experience aura, which is typically visual and precedes the headache onset [2]. Chronic migraines [CMs] can be diagnosed in patients who consistently have at least 15 days of headaches [of which eight have migraine symptoms] per month [3].

Migraines are not a benign disorder and carry increased risk of cardiovascular and cerebrovascular events, especially in premenstrual women [4,5]. Pregnancy appears to be protective, and most women with pre-existing migraines without aura see a significant improvement during pregnancy, and by the third trimester, over 80% are headache-free [6,7]. Migraines, however, have been shown to increase the risk of pregnancy-related complications, including pre-eclampsia and miscarriage [8]. A recent study in a large primary care cohort found that migraines were associated with an 8% higher relative risk of miscarriage, especially in those exposed to triptans [9].

Therapeutic options for the treatment of a migraine in pregnancy and breastfeeding remain limited, as many medicines commonly used for acute treatment and prevention are contraindicated [10]. Topiramate, for example, has been shown to increase the risk of malformation by up to 9% [11]. Of the migraine preventatives approved by the National Institute for Health and Clinical Excellence (NICE), only Propranolol and Amitriptyline are deemed safe during pregnancy [12]. Approximately a third of patients with migraines do not benefit from standard migraine preventatives (including Amitriptyline and Propranolol) and require specialist migraine treatments, including Calcitonin Gene-Related Peptide (CGRP) therapies or OnabotulinumtoxinA (OnabotA) [13].

OnabotA, a neurotoxin produced by the anaerobic *Clostridium botulinum*, has numerous medical indications [14] and over the past three decades has emerged as a specialist treatment for chronic migraines [15]. In addition to causing transient paralysis at the neuromuscular junction, OnabotA also exerts antinociceptive properties and, in the case of migraines, is thought to reduce peripheral and central sensitisation [15]. Its use in migraines was initially proposed following observations in patients receiving aesthetic OnabotA [16], and in 2006, the PREEMPT study demonstrated a significant reduction in monthly headache days compared to placebo in CMs [17]. In this seminal study, participants received 12 weekly injections of OnabotA (31 × 5 units, 155 units total) into the scalp, neck and shoulders and at 24 weeks had on average 8.4 days fewer monthly headache days, compared to 6.6 days with the placebo.

Since PREEMPT [17], real-world data have demonstrated long-term effectiveness and tolerability of OnabotA in patients with CMs [18], and in 2012, NICE approved its use in CMs [19]. At present, OnabotA is not approved by the Medicines and Healthcare Products Regulatory Agency (MHRA) for use during pregnancy or in women of childbearing age who are not using contraception, owing to the inadequate safety data [20,21]. Importantly, at-risk groups were not recruited to the original PREEMPT trial [17], and to date, no trials have addressed the safety of OnabotA in these groups.

Despite robust clinical evidence for its efficacy and tolerability in patients with CMs, the use of OnabotA in pregnancy and breastfeeding remains uncertain. We conducted a systematic review to assess the safety of OnabotA in these groups.

## 2. Methods

### 2.1. Search Strategies

**Figure 1:**
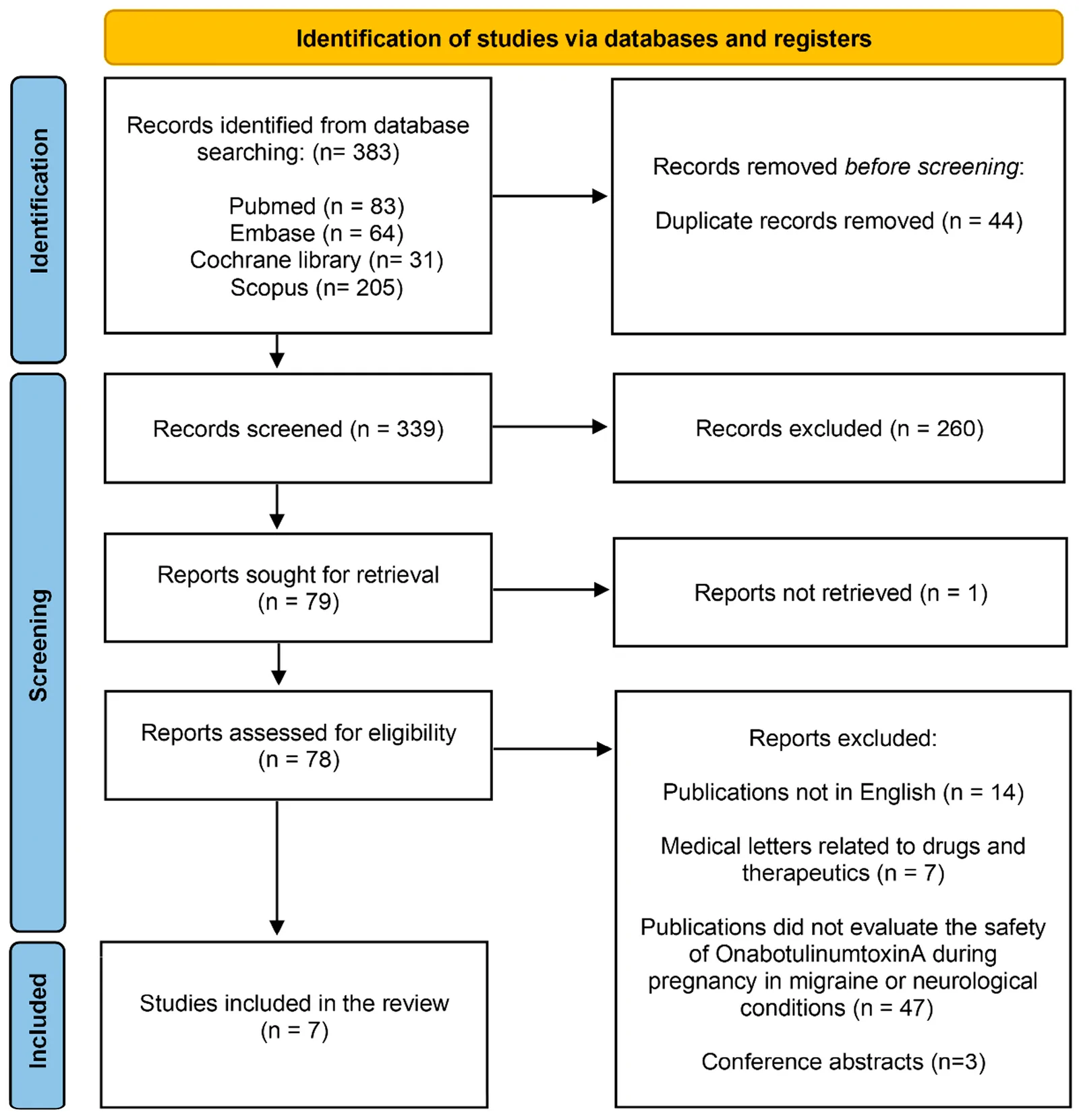
PRISMA—Identification of studies via databases and registers.

A comprehensive literature review was conducted using Medline, Scopus, Cochrane Library, and Embase databases from January 2000 to December 2025 to identify relevant articles, in accordance with Preferred Reporting Items for Systematic Reviews and Meta-Analyses (PRISMA) guidelines [22,23] (Figure 1). The search results were imported into a reference manager and were reviewed by two independent reviewers (AN and EL), and a third reviewer (SE) was consulted in cases of discrepancies. The databases were last reviewed in March 2026. The review protocol has been registered with the International Prospective Register of Systematic Reviews (PROSPERO) [24].

Due to a lack of data exclusively focusing on chronic migraines and neurological indications, a deviation from the original registered protocol was necessary. Specifically, the inclusion criteria were expanded to include a large case series which reported patients receiving OnabotA for multiple indications, including migraines, other neurological conditions and aesthetic purposes. Excluding this critical study would have resulted in a significant loss of available safety and outcome data, thereby compromising the statistical power of this review.

The search strategy included keywords related to migraines, other neurological conditions, aesthetic applications, OnabotulinumtoxinA (OnabotA), and pregnancy-related outcomes. The detailed search strategies for each database are provided in the Supplementary File.

### 2.2. Eligibility Criteria

Inclusion criteria were defined according to the PICOS (Patients, Intervention, Comparison, Outcomes, and Study design) framework [25]. We included studies involving patients diagnosed with chronic migraines and other neurological conditions (e.g., dystonia), who received OnabotA during preconception (3 months prior), during pregnancy, and/or in the postpartum period if breastfeeding.

The exclusion criteria were studies not primarily published in English; studies with unknown pregnancy outcomes; publications without primary data; case series which did not include migraines or other neurological conditions; conference abstracts; and letters.

### 2.3. Data Extraction

Data extracted from studies included the study title, publication year, authors, study design, indication for OnabotA injection, number of patients included, number of pregnancies and foetuses, timing of OnabotA injection in relation to the pregnancy stage, and pregnancy outcome.

### 2.4. Outcome

The outcomes of interest included (i) foetal loss; (ii) foetal anomalies, which were categorised [26] as minor congenital anomalies (primarily cosmetic or rarely medically significant), major congenital anomalies (with medical and/or social implications often requiring surgical intervention), birth complications (occurring during labour or delivery), or genetic abnormalities (including pathogenic sequence variants or chromosomal abnormalities); and (iii) neurodevelopmental outcomes during breastfeeding, including neuromuscular, developmental, or cognitive impairments.

### 2.5. Risk of Bias Assessment

Risk of bias was assessed by two independent reviewers (AN and EL), and a third reviewer (SE) was consulted in cases of discrepancies. The cohort studies were evaluated using the ROBINS-I tool. Survey and case reports were evaluated narratively, as ROBINS-I is not applicable to these study designs.

**Figure 2:**
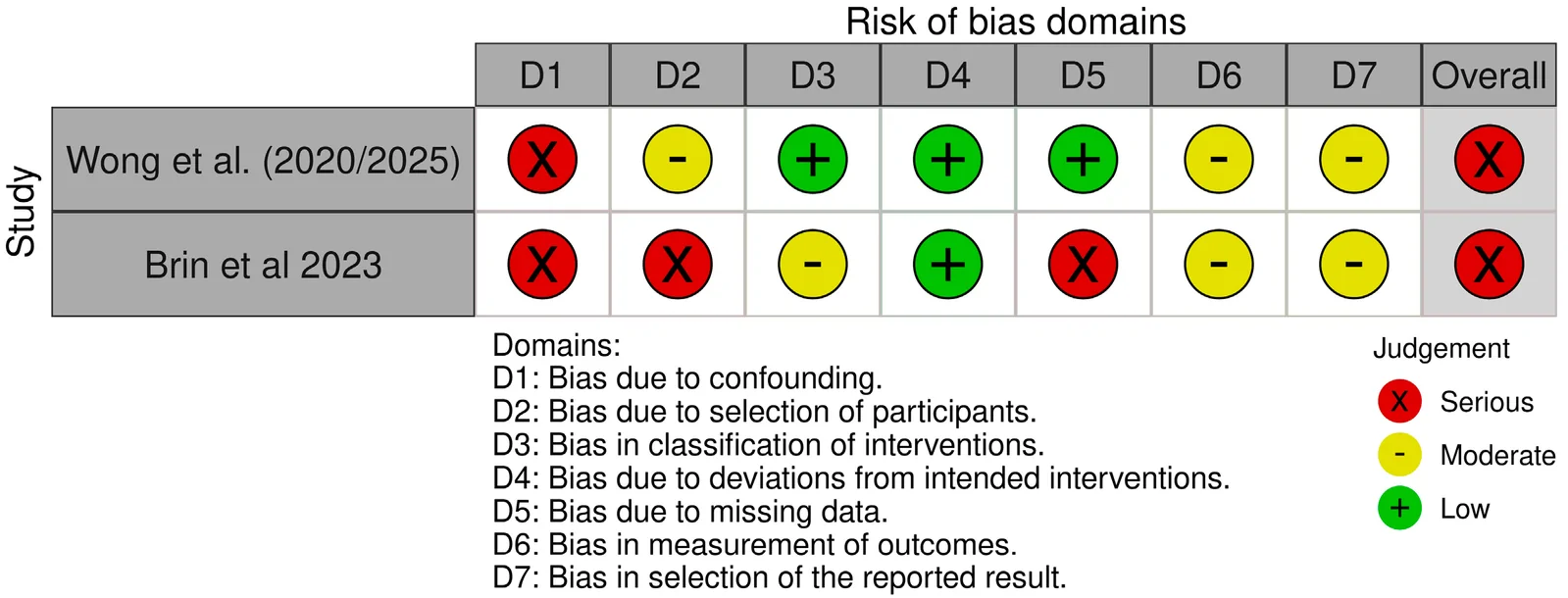
Detailed risk-of-bias assessment [27,28,29].

**Figure 3:**
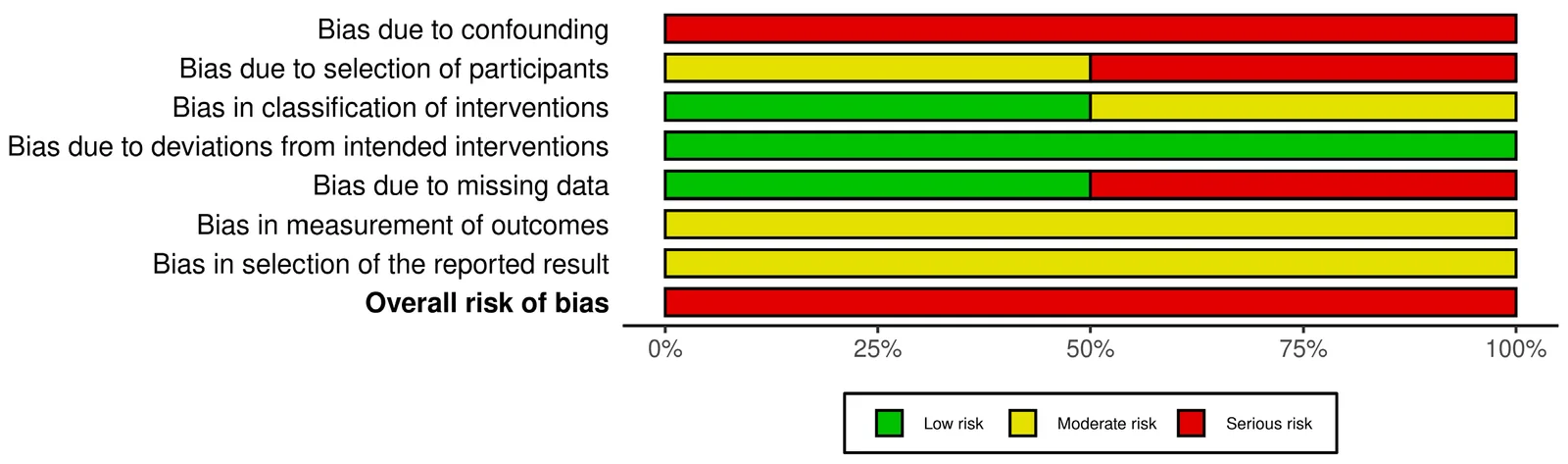
Summary percentages of risk-of-bias judgements.

The cohort studies by Brin et al. [27] and Wong et al. [28,29] were judged to be at high risk of bias due to confounding, participant selection, and the absence of a control group (Figure 2 and Figure 3). In Wong et al.’s research [28,29], continuation of OnabotA treatment during pregnancy was determined by patient preference, introducing self-selection bias. Brin et al. [27] were considered to have a serious risk of bias because of their reliance on retrospective and spontaneous reports, substantial missing exposure data, and manufacturer-sponsored data collection, all of which increased the risks of selection, information, and reporting bias.

The survey by Morgan et al. [30] was considered to be at high risk of recall bias, self-selection bias, and reporting bias. The third question in the survey was “Approximately how many patients have you injected with Botox?”, relying on estimated rather than verified figures. This introduces measurement imprecision and reduces the reliability of the reported data.

The case reports by Newman et al. [31], Robinson et al. [32], and Mezaal et al. [33] were deemed to be at high risk of bias due to their uncontrolled design, susceptibility to publication bias, selection bias, and limited generalisability.

## 3. Results

A total of 383 articles were identified. After removing duplicates, 339 remained for screening. Of these, 260 were excluded based on the inclusion and exclusion criteria, leaving 79 eligible for full screening; after full screening, seven articles were included in the review.

The current evidence base is predominantly observational, consisting of one large postmarketing safety database analysis that included both prospective and retrospective pregnancy reports [27], two cohort-like real-world series from a tertiary headache centre [28,29], one physician survey [30], and three case reports [31,32,33] (Table 1). Across the available studies, most exposures occurred either in the preconception period or during the first trimester, and most data related to OnabotA rather than other Botulinum toxin preparations. Overall, the evidence base was limited by small sample sizes, descriptive designs, incomplete ascertainment of outcomes, and likely reporting bias.

A total of 542 women were identified as having received OnabotA during pregnancy, based on analyses of seven studies: three cohort studies, three case reports, and one survey [27,28,29,30,31,32,33]. Some women experienced multiple pregnancies or twin pregnancies, bringing the total number of pregnancies to 548, and the total number of foetuses to 555. Of the 542 patients included in this review, indications included migraines, 228 (42%); aesthetic, 120 (22%); other neurological conditions (e.g., dystonia), 97 (18%); and non-neurological conditions (e.g., hyperhidrosis), 40 (7%). In 57 cases (11%), the indication was unknown.

The timing of OnabotA treatment was analysed: 101 received doses during the pre-conception period, 344 during the first trimester, 17 in the second trimester, and 10 during the third trimester. The injection timing was unknown in 81 cases. OnabotA doses ranged between 1.25 Units and 400 units.

- **Foetal loss**

Data on foetal loss were available from seven observational studies [27,28,29,30,31,32,33], comprising 542 pregnancies and 555 foetuses with reported outcomes. Foetal loss was reported in approximately 17% of pregnancies.

The largest dataset was the 29-year Allergan global safety database analysis [27], in which 92/404 (22.8%) foetuses were lost across both prospective and retrospective reports. Among 195 prospective pregnancies, 45/197 (22.8%) foetuses were lost, comprising 32 spontaneous losses and 13 elective abortions. In the retrospective cohort, 47/207 (22.7%) pregnancies ended in foetal loss, including 40 spontaneous losses and seven elective abortions. Because retrospective reports are prone to over-reporting of adverse outcomes, the prospective data are likely more informative, although even these data were derived from passive safety reporting. A more recent real-world migraine cohort reported lower absolute numbers of foetal loss. In the updated 2025 cohort [29] (which included all data from 2020 [28]) of 126 pregnancies, a miscarriage occurred in 2/97 (2.1%) women who continued treatment and 1/29 (3.4%) who discontinued treatment; no obvious cause for miscarriage was identified in these cases. Three small case reports and one physician survey showed low rates of miscarriage or elective termination [although this may have been for unrelated reasons] [30,31,32,33].

In summary, although miscarriage and foetal loss were reported across studies, no consistent signal of increased risk following OnabotA exposure was identified. However, the certainty of this evidence is low due to predominantly small, uncontrolled, and descriptive studies.

- **Foetal anomaly**

Data on foetal anomalies were available from seven observational studies [27,28,29,30,31,32,33], comprising 458 live births with reported outcomes.

The largest study reported 152 prospective live births [27], of which 148 (97.4%) were normal and four (2.6%) were associated with abnormalities, including one major congenital anomaly [prevalence of 0.7%] and three minor anomalies or birth complications. In the retrospective cohort [27], foetal anomalies were reported in 5/160 (3.1%) live births; however, these data were not used to estimate prevalence because of the inherent risk of reporting bias. In contrast, smaller real-world cohort studies [28,29] and case-based reports [30,31,32,33] did not identify congenital anomalies, although these studies were limited by small sample sizes and incomplete ascertainment.

The available evidence does not demonstrate a clear signal for increased risk of foetal anomalies following maternal exposure to OnabotA. However, the certainty of this evidence is low, owing to the limited number of studies, small sample sizes, and reliance on observational and passive reporting data, which may not detect rare or long-term outcomes.

- **Breastfeeding**

Data on neuromuscular development during breastfeeding were very limited, with evidence only available from one real-world case series [29]. Here, 80/97 (82.5%) women who continued treatment breastfed. Of the 29 women who discontinued treatment, 20 breastfed (69.0%). No adverse developmental outcomes were reported up to 1 year.

Overall, there was insufficient evidence to determine the safety of OnabotA exposure during breastfeeding with respect to infant neuromuscular development.

## 4. Discussion

This systematic review provides a comprehensive up-to-date analysis of pregnancy-related complications associated with exposure to OnabotA in the treatment of neurological conditions, principally CMs.

In summary, of the 555 OnabotA-exposed pregnancies, 80.2% resulted in full-term births, with a 17.5% loss rate (73 spontaneous, 21 elective, three unspecified) and 1.6% reporting foetal anomalies. These findings align with general population baselines for miscarriages (12.5–18.7%) and foetal anomalies (3–5%), respectively [34,35]. Among 94 cases of reported breastfeeding, no adverse infant events or developmental delays were observed at 1 year. Maternal outcomes showed favourable symptom control without adverse effects.

- **Considerations from botulism in human pregnancies and animal models**

Non-medicinal systemic exposure to botulinum toxin (e.g., through foodborne contamination or via wound infection), even in small amounts, may result in botulism, a life-threatening neurological emergency, characterised by symmetrical descending paralysis in the absence of sensory deficits. Six cases of botulism during pregnancy have been reported [36,37,38,39,40]. In all cases, botulism was confirmed in the mother, but none of the neonates showed direct clinical or laboratory evidence of botulism. Healthy full-term deliveries with no adverse developmental sequelae were observed in five cases [37,38,39,40]. One case resulted in developmental delays, which were attributable to perinatal hypoxia and haemorrhage rather than botulinum toxicity [36].

Animal studies investigating the effects of OnabotA during pregnancy are limited and showed mixed results depending on dosage, frequency of administration, site of administration, species and timing of exposure. Lethal dose intravenous administration of OnabotA in pregnant rabbits [41] did not result in detectable levels of the toxin in placental or foetal tissues at the time of maternal death. In rats, daily 0.125–8 units/kg intramuscular dosing during organogenesis resulted in significant maternal toxicity, premature delivery, spontaneous abortions, and maternal death [20,21]. Less toxicity was observed when 4–16 units/kg was administered only twice during organogenesis [20,21]. Together with reports of botulism during human pregnancy [36,37,38,39,40], these findings suggest that botulinum toxin is unlikely to cross the placenta, even when present at clinically significant levels in the maternal circulation. Furthermore, this is biologically plausible, as botulinum toxin is a large protein molecule (approximately 150 kDa), which limits passive placental transfer.

Limited data exists regarding the transfer of OnabotA to breast milk. Following intramuscular administration at recommended doses, OnabotA does not appear to enter the systemic circulation [42] or breast milk at significant concentrations [43,44].

The pharmacodynamics of therapeutic OnabotA dosing should also be considered. Although the non-metabolised half-life of OnabotA is relatively short [a few minutes], its biological effects may persist for up to 5 months [45] and therefore therapeutic dosing is typically administered at 12-week intervals. Furthermore, the 16 units/kg used in some animal studies is substantially higher than the equivalent therapeutic doses used in humans. Collectively, these data suggest that localised administration of OnabotA at therapeutic doses at 12-week intervals is associated with a low theoretical risk to the foetus during pregnancy and breastfeeding.

### 4.1. Strengths and Limitations of This Systematic Review

An important strength of this review is the comprehensive search across multiple databases and the large number of pregnancies included. A total of 542 pregnant women were identified as having received OnabotA during pregnancy, of whom 228 received it for migraines, making it the most extensive study to date analysing the safety of OnabotA as a migraine preventative in pregnancy. The rarity of the intervention makes the number of eligible pregnancies identified particularly relevant. This review spans 25 years, from before the approval of OnabotA for CMs, and covers various study designs, including cohort studies, case reports, and surveys. Finally, detailed reporting of foetal outcomes, contextualised with prevalence data, enables a relevant interpretation of the results.

This review also has some important limitations, given the rarity of the targeted population and the paucity of available data. This review lacks high-quality evidence, relying primarily on observational studies, expert opinion, and anecdotal experience, with no randomised controlled trials. Data on breastfeeding were very limited and could only be assessed from a single study [29]. Furthermore, the majority of pregnancy data were derived from a single manufacturer-sponsored post-marketing safety database [28] and therefore substantially influenced the overall findings. Although this does not invalidate the available evidence, the findings should be interpreted with appropriate caution, given the potential for reporting and selection biases. Other limitations include the variability in doses and indications, which introduce confounding variables and limit the dose–response analysis, thereby limiting the statistical analysis of cause and effect, and the generalizability of the pooled safety estimates to women with chronic migraines. This was most obvious in the largest cohort by Brin et al. [28], where subgroup analysis of patients with migraines was not possible. As with all retrospective studies, this review is subject to selection bias, as only 228 subjects are an accurate representation of the pre-identified population, and to recall bias, as the analysed data are based on retrospective recall from one of the eligible studies.

### 4.2. Future Directions

This systematic review highlights the need for future research into the safety of OnabotA in pregnancy and breastfeeding. Although randomised controlled trials are unlikely to be feasible or ethical in this setting, well-designed prospective observational studies could substantially strengthen the current evidence base. This could be achieved with prospective multicentre pregnancy registries with standardised reporting of treatment indication, dose, timing of exposure, maternal characteristics, pregnancy outcomes, congenital anomalies, and long-term infant neurodevelopment. Where feasible, future studies should also include pregnant women with chronic migraines who were not exposed to OnabotA during pregnancy, to better determine whether OnabotA modifies the baseline pregnancy risks associated with a migraine itself. Furthermore, given the rarity of therapeutic OnabotA exposure during pregnancy, collaboration between different specialities (e.g., neurologists, dermatologists, urologists) will be essential to establish sufficiently powered registries and generate more robust safety data.

## 5. Conclusions

Although there is limited evidence available on the use of OnabotA during pregnancy for the treatment of chronic migraines, and the existing evidence is derived predominantly from observational studies, no demonstrable increase in the risk of adverse maternal or foetal outcomes has been identified. Evidence regarding breastfeeding is even more limited. However, among the reported cases, no adverse infant neuromuscular or developmental outcomes were observed during the 1 year of follow-up. These findings should be interpreted with caution, given the limited quality and heterogeneity of the available evidence. OnabotA may therefore be considered on a case-by-case basis in carefully selected women with chronic migraines (and other neurological conditions) when safer or established alternatives are ineffective, contraindicated, or not tolerated.

## Figures and Tables

**Table 1:** A summary of results from eligible studies.

Study	Number of Patients	Number of Pregnancies	Number of Foetuses	OnabotA Dose/U	Number of Doses *	Indication (*n*)	Timing of Injection (*n*)	Pregnancy Outcomes			Lactation
								Full Term—Healthy	Foetal Anomalies/ Abnormal Outcomes in Live Births	Foetal Loss (*n*)	
Brin et al. [27]	397	397	404	<50–>400	N/A	Aesthetic (120) Migraines (100) Headache (3) Movement Disorders (41) Hyperhidrosis (27) Urological Disorders (7) Gastrointestinal Disorders (6) Spasticity (6) Pain disorders (3) Miscellaneous Neurological Disorders (27) Unknown (57)	Preconception (51) 1st trimester (250) 2nd trimester (12) 3rd trimester (5) Unknown (79)	299	Foetal anomalies (9) Premature birth (4)	Miscarriage (72) Abortion (20)	0
Wong et al. [29] **	126	126	126	N/A	N/A	Migraines (126)	Pre-conception (46) 1st Trimester (80) 2nd Trimester (N/A) 3rd Trimester (N/A) Unknown (0)	123	0	3	94
Morgan et al. [30]	16	19	19	1.25–30	N/A	Cervical dystonia (9) Strabismus (2) Blepharospasm (2) Limb dystonia (1) Oromandibular dystonia (1) Spasmodic dystonia (1)	Pre-conception (0) 1st Trimester (12) 2nd Trimester (1) 3rd Trimester (1) Unknown (1)	17	0	Spontaneous (1) Abortion (1)	0
Newman et al. [31]	1	4	4	300	2–4	Cervical dystonia (1)	Pre-conception (4) 1st Trimester (2) 2nd Trimester (3) 3rd Trimester (4) Unknown (0)	4	0	0	0
Robinson et al. [32]	1	1	1	71	1	Migraines (1)	Pre-conception (0) 1st Trimester (0) 2nd Trimester (1) 3rd Trimester (0) Unknown (0)	1	0	0	0
Mezaal et al. [33]	1	1	1	155	N/A	Migraines (1)	Unknown (1)	1	0	0	0

* number of doses given during the pre-conception period and pregnancy, and ** the updated study published in 2025.

## Data Availability

No new datasets were generated. All analyzed data are included within this article and its Supplementary Materials.

## Supplementary Materials

The following supporting information can be downloaded at https://www.mdpi.com/article/10.3390/life16081228/s1. PRISMA 2020 checklist.
